# Physicians discuss the risks of smoking with their patients, but seldom offer practical cessation support

**DOI:** 10.1186/s13011-015-0039-9

**Published:** 2015-11-02

**Authors:** Jaana Keto, Jari Jokelainen, Markku Timonen, Kari Linden, Tero Ylisaukko-oja

**Affiliations:** Department of General Medicine, Faculty of Medicine, University of Oulu, P.O. Box 5000, FIN-90014 Oulu, Finland; Unit of General Practice, Oulu University Hospital, FIN-90014 Oulu, Finland; Pfizer Oy, Tietokuja 4, FIN-00330 Helsinki, Finland

**Keywords:** Smoking, Smoking cessation, Attitudes, Primary care, Secondary care, Nicotine replacement therapy, Withdrawal medication, Nicotine

## Abstract

**Background:**

Our aim was to study the smoking cessation-related 1) attitudes & experiences and 2) consultation practices of Finnish physicians and to determine if there is a relationship between the two.

**Methods:**

An online survey on smoking cessation was sent to 39 % of all Finnish physicians, with emphasis on physicians working in fields relevant to smoking cessation. A total of 1141 physicians (response rate 15 %) responded to the online survey, 53 % of whom were employed in primary health care. A total of 1066 respondents were eligible for the analysis. The questionnaire included questions on the physician’s own smoking status, their attitudes and experiences on smoking cessation, and the implementation of smoking cessation in clinical practice. Two sub-scales concerning smoking-related consultation activities were constructed: one for conversation, and another for practical actions.

**Results:**

The most common consultation activities (respondents who reported doing the following actions “nearly always”) were asking how much the patient smokes (65 %), marking smoking status in patient records (58 %) and recommending quitting to the patient (55 %). The least common activity was prescribing withdrawal medication (4 %). Primary care physicians were more active than those working in secondary health care in nearly all activities mapped. A positive attitude and experiences on smoking cessation were associated with actively offering withdrawal support. Those who were familiar with the local treatment guidelines for tobacco addiction were 30 % more active in offering practical cessation help to their patient. The respondents were more active in discussing smoking with their patients than in offering practical cessation help.

**Conclusion:**

Physicians offer their patients practical cessation support relatively infrequently. Practical cessation calls for continuous education of physicians about the nature of tobacco and nicotine addiction, the role of smoking as a risk factor for various diseases, and the practical measures needed for smoking cessation. Secondary care physicians should acknowledge the authority they pose toward smoking patients.

## Background

Smoking continues to be the most significant preventable cause of death in the Western world, causing a fifth of all deaths [1] and reducing life expectancy by 10 years [2]. While the majority of smokers want to quit, only about one third of them receive help from a physician [3, 4]. However, seeking help from a physician greatly raises the odds of a smoker’s success with cessation. A 3–10-min discussion with a physician raises the likelihood for the patient’s success in quitting by 1.6-fold, and even up to 2.3-fold if the discussion exceeds 10 min in length [3].

When the efficacy of different withdrawal methods has been evaluated, combining behavioral support with pharmacotherapy has proven to be the most effective form of smoking cessation aid [3–5]. It is thus of key importance that a physician not only recognizes the adverse health effects of smoking and is aware of the smoking status of their patient, but also knows how to support the patient.

Previous studies have shown that physicians are increasingly active in recommending quitting to their smoking patients. However, when it comes to aiding the patients in their cessation attempts in practice, the activity rates drop dramatically [6–8]. In this study, we took a closer look at this phenomenon from the physician’s perspective. We set out to not only map 1) physicians’ attitudes and experiences on smoking cessation and 2) their smoking cessation activities, but also to test whether there is an association between the two. From earlier studies we know, for instance, that physicians who smoke are less likely to initiate cessation interventions in comparison to their non-smoking colleagues [9]. In addition, we analyzed how smoking-related consultation activities are carried out in primary healthcare in comparison to secondary health care. The objective was to collect information that could be used in targeted physician training and motivation, which will ultimately lead to smoking cessation that is more effective for the patient and more rewarding for the physician.

## Methods

### Participants

An invitation to answer an online questionnaire on smoking cessation was sent to a random sample of 7830 Finnish physicians in December 2012, covering 39 % of all Finnish physicians at the time. The invitation was sent to physicians whose e-mail address was available for research projects in the membership register of the Finnish Medical Association. The sample covered both general practitioners and specialists from fields relevant to smoking cessation. The specialists that we targeted worked in general practice, occupational health care, obstetrics and gynaecology, surgery, respiratory diseases and allergology, internal medicine (covering, amongst others, hematology, infectious diseases and cardiology), psychiatry, and oncology. The respondents who did only administrative, research or other non-clinical work were excluded from the analyses. In the subset of analyses, primary care physicians and secondary care physicians were analyzed as separate groups.

### Electronic data collection

The total number of targeted physicians was 7830 and data collection consisted of three rounds of e-mail invitations. The total number of physicians who entered the survey was 1390, of whom 1141 (82.1 %) completed the survey. Thus, 15 % of those invited to participate in the study completed the survey. The Webropol online survey tool was used for data collection (www.webropol.com).

### Questionnaire and consultation sub-scales

The study questionnaire included questions about the respondent’s demographics, smoking status, attitudes and experiences on smoking and smoking cessation, implementation of smoking cessation in clinical practice, barriers in smoking cessation, and familiarity with the Finnish treatment guidelines for tobacco addiction and smoking cessation.

**Fig. 1:**
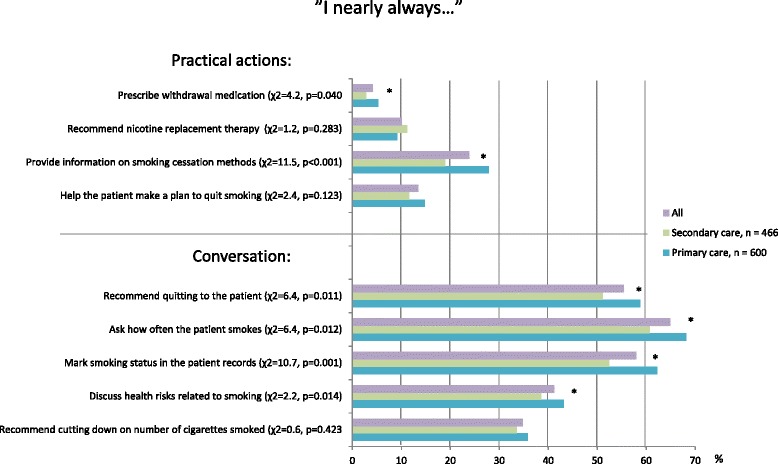
Smoking cessation help offered by Finnish physicians. Percentages of respondents who reported taking the following actions “nearly always”. *n* = 1066, df = 1 for all items, * = *p* < 0.05 (*χ* ^2^ test)

The questions on consultation activities were chosen from the well-known study by Pipe and colleagues to allow international comparison [7]. The activities were also in line with local clinical guidelines for smoking cessation [5]. There were a total of 10 items, for which a four-point grading system was applied: “nearly always” (3), “often” (2), “sometimes,” (1) and “never” (0). The consultation items were divided into two categories based on statistical and content-related analysis: conversation and practical actions. “Conversation” covered behavior that acts as a mini intervention; sending the patient a message that smoking is something the physician is deeply concerned about. “Practical actions” covered items that make it easier for the patient to quit once they have made the decision to do so, such as helping the patient make a quitting plan or offering pharmaceutical cessation aid. Pharmaceutical aid can be either over-the-counter nicotine replacement therapy or prescription medication (bupropion, varenicline, nortriptyline). These activities are listed in Fig. 1. The scores gained in the consultation sub-scales were utilized when searching for an association between consultation activities and smoking-related attitudes and experiences.

The scale for smoking-related attitudes and experiences was as follows: “completely agree,” “somewhat agree,” “somewhat disagree,” or “completely disagree.” Physicians answering either “completely” or “somewhat agree” were combined as an “agree” group, whereas the remaining respondents were included in the “disagree” group. The question concerning the Finnish treatment guidelines for smoking cessation was graded as follows: “familiarized myself thoroughly,” “familiarized myself in outline,” “browsed through,” “heard about the guideline but did not read,” and “do not know.” Physicians responding “familiarized myself thoroughly” and “familiarized myself in outline” were included in the “agree” group, and the others in the “disagree” group.

### Statistical analysis

Distributions of continuous variables were expressed as mean and standard deviation (SD), and categorical variables as proportions. Pair-wise comparisons of continuous variables between groups were tested using the Mann–Whitney *U*-test (MW-U), and categorical data was tested with *χ*^2^ or Fisher’s exact test, as appropriate.

Exploratory principal components analysis (PCA) was initially used to explore the dimension structure of the consultation activities. Promax rotation was applied. The scree plot and total-variance-explained variability criteria were used to specify the retained factor. This analysis produced two sub-scales: a) the conversation scale (5 items; each scored from 0 to 3) and b) the practical actions scale (4 items; each scored from 0 to 3). The action “refer patient to another health care provider, such as a nurse or specialist clinic” that was mapped in the survey remained alone in the PCA analysis, and was therefore excluded from the two sub-scales. The total variance explained was 72 %. A polychoric correlation matrix was used in the PCA. Reliability of the factor solution was determined by calculating internal consistency using Cronbach’s alpha with a corresponding 95 % confidence interval (CI).

All statistical tests were two-tailed, and *p*-values < 0.05 were considered statistically significant. Statistical analysis was performed using the R software environment, version 3.0.0 (R Core Team, 2013).

## Results

### Participants

An overview of the study participants (*N* = 1141) is presented in Table 1. The distributions of age, sex, geographical location and specialty corresponded to the Finnish base of physicians (data on file) [10]. A total of 2.2 % (3.5 % male, 1.2 % female; total *n* = 25) of the participants smoked daily and an additional 5.3 % (7.9 % male, 3.3 % female; total *n* = 60) were occasional smokers.

**Table 1:** Description of the study sample

Description	n (%)
Place of work:	
Primary health care	600 (52.6)
Secondary health care	466 (40.8)
Other (non-clinical work)^a^	75 (6.6)
Total	1141 (100)
Specialists vs general practitioners:	
General practitioner	126 (11.0)
Specialist	1015 (89.0)
Gender:	
Male	481 (42.2)
Female	660 (57.8)
Smoking status:	
Daily smoker	25 (2.2)
Occasional smoker	60 (5.3)

^a^Removed from analysis

### Consultation sub-scales

The two consultation sub-scales (conversation and practical actions) constructed showed good internal consistency. The Cronbach’s alpha for the conversation subscale was 0.79 (95 % CI 0.77–0.81) and 0.80 (95 % CI 0.78–0.82), for the practical actions scale. The correlation coefficient between the conversation and practical action scales was 0.611 (df = 1064, *p* < 0.001).

### Consultation activities

Physicians were more active in discussing smoking with their patients than in offering practical support and tools for quitting. The most common consultation activities (respondents who reported doing the following “nearly always”) were asking how much the patient smokes (65 %), marking smoking status in patient records (58 %) and recommending quitting to the patient (55 %). The least common activities were prescribing withdrawal medication (4 %) and recommending nicotine replacement therapy (10 %) (Fig. 1).

Primary care physicians were more active than secondary care physicians in most individual consultation activities (*χ*^2^ and *p*-values in Fig. 1).

### Attitudes and experiences on smoking cessation

**Fig. 2:**
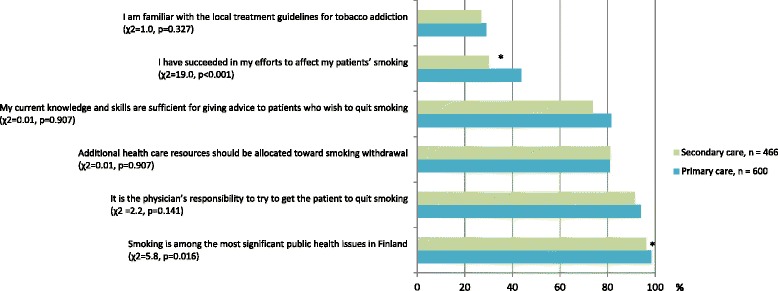
Smoking cessation related attitudes and experiences of Finnish physicians. *n* = 1066, df = 1 for all items, * = *p* < 0.05 (*χ* ^2^ test)

Most of the participants agreed that smoking is among the most significant public health issues in Finland (97.3 %), that it is the physician’s responsibility to try to get the patient to quit smoking (92.8 %), and that additional health care resources should be allocated to smoking cessation (81.0 %) (Fig. 2).

A total of 78.0 % of respondents considered their current knowledge and skills to be sufficient for giving advice to patients who wish to quit smoking. However, only 37.7 % of physicians said that they have succeeded in their efforts to affect their patients’ smoking. The difference in attitudes and experiences between primary and secondary care physicians was relatively small, and reached statistical significance for only two items (*χ*^2^ and *p*-values in Fig. 2).

### The effect of positive smoking cessation related attitudes and experiences on the cessation support given

**Fig. 3:**
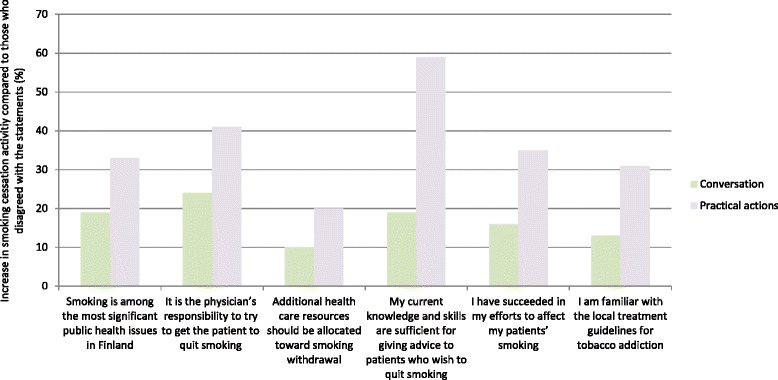
The effect of positive attitudes and experiences on smoking cessation activity. The increase in smoking cessation activity of physicians who agree with the claims presented compared to those who disagree. The baseline (0 %) is the activity level of physicians who disagree with the claims. For instance, those who are familiar with the local treatment guidelines for tobacco addiction are 30 % more active in offering practical cessation help to their patient. *p* < 0.005 (Mann–Whitney *U*-test) for all items, *n* = 1066

The relationship between 1) smoking cessation related attitudes and experiences and 2) consultation activity is presented in Table 2. A statistically significant positive association was seen with all attitude claims presented (MW-U, Z- and *p*-values in Table 2) (Fig. 3). The strongest association was observed between the respondents’ consultation activity and their evaluation of their own withdrawal skills. Physicians who found their skills sufficient for giving withdrawal aid were more active measured on both the conversation scale (+20 %) and the practical actions scale (+60 %) than those who considered their skills insufficient. The same applied to being familiar with the local treatment guidelines for tobacco addiction (+14 % more active on conversation and +29 % more active on practical actions scale).

**Table 2:** The relationship between consultation activity and smoking-related attitudes and experiences

Claim	Conversation, score 0-15			Practical actions, score 0-12		
	N Mean score (SD)			N Mean score (SD)		
	Agree	Disagree	MW-*U* test	Agree	Disagree	MW-*U* test
Smoking is among the most significant public health issues in Finland	1037 **11.6** (2.9)	29 **9.7** (3.4)	Z = −3.2, *p* = 0.002	1037 **6.1** (2.5)	29 **4.6** (2.1)	Z = −3.3, *p* < 0.001
It is the physician’s responsibility to try to get the patient to quit smoking	989 **11.7** (2.8)	69 **9.4** (3.7)	Z = −5, *p* < 0.001	989 **6.2** (2.5)	69 **4.4** (2.4)	Z = −5.7, *p* < 0.001
Additional health care resources should be allocated toward smoking withdrawal	863 **11.7** (2.8)	203 **10.7** (3.2)	Z = −4.3, *p* < 0.001	863 **6.3** (2.5)	203 **5.2** (2.3)	Z = −5.4, *p* < 0.001
My current knowledge and skills are sufficient for giving advice to patients who wish to quit smoking	832 **11.9** (2.6)	233 **10.0** (3.5)	Z = −7.8, *p* < 0.001	832 **6.6** (2.3)	233 **4.2** (2.4)	Z = −12.3, *p* < 0.001
I have succeeded in my efforts to affect my patients’ smoking	402 **12.7** (2.2)	534 **11.0** (2.8)	Z = −9.9, *p* < 0.001	402 **7.4** (2.2)	534 **5.5** (2.3)	Z = −12.3, *p* < 0.001
I am familiar with the local treatment guidelines for tobacco addiction	300 **12.6** (2.4)	766 **11.1** (3.0)	Z = −7.5, *p* < 0.001	300 **7.3** (2.2)	766 **5.6** (2.5)	Z = −10.1, *p* < 0.001

Consultation activity is divided into conversation and practical actions as explained in Methods. Bold data refers to the Mean score

## Discussion

### Principal findings

Physicians are more active in discussing smoking with their patients than in offering practical support and tools for cessation. Primary care physicians are more active in giving smoking-related consultation than those working in secondary health care. The most frequently performed smoking cessation-related activities are 1) asking how often the patient smokes, 2) marking smoking status in patient records and 3) recommending quitting to the patient. Consultation activity was associated with positive attitudes and experiences on smoking cessation.

### Strengths and limitations of the study

The study consisted of a large pool of 1141 Finnish physicians from both primary and secondary health care. The survey covered a wide spectrum of smoking cessation related items, thereby allowing thorough analyses of both the attitudes and the practical aspects of smoking cessation in the Finnish health care system from the physicians’ point of view.

Since we targeted specialists from fields relevant to smoking cessation, respondents who consider the subject of smoking cessation important are likely to be overrepresented in the sample. The reality of smoking cessation attitudes and practices among Finnish physicians may thus be grimmer than our results suggest. Also, earlier studies have shown that physicians tend to paint a more optimistic picture of their smoking cessation practices than their patients do – and this has especially proven to be the case when investigating how often the physician recommends quitting to the patient [4, 11].

Electronic surveys have many advantages over traditional methods, but there are limitations as well, such as generally lower response rates and the respondents’ reduced willingness to use time on the survey [12, 13]. This was also a limitation of the present study: only 15 % of those invited to participate in the study filled in the questionnaire. However, it was not possible to exactly determine how many of the targeted physicians were reached, i.e., how many of them made a conscious decision not to participate in the survey and how many didn’t open the e-mail invitation.

In this study, tobacco use was limited to cigarettes. Addiction to moist snuff, electronic cigarettes or other nicotine products was marginal in Finland when this study took place. It should also be noted that the cessation methods mapped in this study are in line with the local clinical guidelines, which do not recognise electronic cigarettes as a potential smoking cessation aid. As alternative forms of tobacco consumption have grown more popular, future studies on smoking cessation should take them under closer inspection.

### Findings in relation to other studies

Physicians smoke significantly less than the general population, and smoking in the profession has been getting more and more marginal in the past decades [14, 15]. Even when taking this progression into consideration, the smoking prevalence found in the present study – 2 % – is historically low. It is possible that a higher response rate could have raised the observed prevalence slightly. However, given that smoking prevalence among Finnish physicians was only slightly higher in 2006, our result seems plausible [16].

Our results show that practical measures to support the patient with quitting are taken rather infrequently. Similar results were obtained also by Pipe and colleagues in their international survey, as well as in several other studies [5–8]. One explanation could be that physicians aren’t aware of the methods available. It is noteworthy that more than 20 % of our respondents found their smoking cessation skills and knowledge inadequate. A systematic review of research done in different countries on the same subject arrived at a similar result [17]. Our association analysis showed that insecurity in one’s skills, along with being unfamiliar with treatment guidelines for tobacco addiction, went hand-in-hand with being passive in offering practical support to patients who want to quit smoking.

Primary health care physicians seem to perform tobacco withdrawal-related consultation more systematically than those working in secondary health care. This has also been demonstrated in another Finnish study [18]. The difference in the base of patients and the nature of appointments may partially explain this phenomenon. However, although smoking cessation is more pronounced in the work of primary health care physicians, it is important that the specialists working in secondary health care are also familiar with support tools. It is well known, for example, that continued cigarette smoking after percutaneous transluminal coronary angioplasty significantly increases the risk of restenosis [19]. There are numerous similar examples in other fields of medicine as well. Even if a secondary care physician recognises their role in supporting the patient with quitting, they may neglect to bring up the issue because of perceived lack of time [17, 20]. However, even a brief and casual intervention from an authoritative secondary care physician can be highly influential on a patient in a receptive state of mind [3, 21]. A hospital patient may already be contemplating on their health and lifestyle at the time of the visit; if a physician utilises this opportunity to express their concern of the patient’s smoking and link it to the patient’s state of health, the effect may be even greater than if the intervention were performed at a standard health check-up.

## Conclusions

Finnish physicians consider smoking to be a major public health problem, yet they relatively seldom offer patients practical cessation support. The better acquainted the physician is with clinical guidelines and methods available for smoking cessation, the more likely they are to actively help their patient with quitting. This calls for continuous education of physicians about the practical measures needed for successful smoking cessation. The physician should be familiar with both non-pharmaceutical and pharmaceutical methods, as the combination of the two has been proven to be most effective [3–5]. Using effective methods is also a significant motivator for the physician, as we found that physicians with successful past interventions were more active in the present as well, thus creating a virtuous circle of effective smoking cessation work. Increased awareness of methods of successful smoking cessation and physician’s role in it is needed especially in secondary health care.

## Key points

- Physicians often discuss smoking with their patients, but practical measures to help the patient quit are taken less frequently.
- Primary care physicians are more active in smoking cessation than their secondary care colleagues.
- The most frequently given smoking cessation consultations are 1) asking how much the patient smokes, 2) marking smoking status in the patient records and 3) recommending quitting to the patient.
- Physicians’ positive attitudes and experiences with smoking cessation, alongside with familiarity to clinical treatment guidelines, are significantly related to how actively they offer cessation support.
